# Prognostic significance of pretherapeutic body mass index in surgically treated oral squamous cell carcinoma

**DOI:** 10.3389/fonc.2025.1686528

**Published:** 2025-11-17

**Authors:** Maximilian Richter, Christian Doll, Friedrich Mrosk, Elena Hofmann, Steffen Koerdt, Max Heiland, Konrad Neumann, Korinna Jöhrens, Jan-Dirk Raguse

**Affiliations:** 1 Department of Oral and Maxillofacial Surgery, Charité - Universitätsmedizin Berlin, Corporate Member of Freie Universität Berlin and Humboldt-Universität zu Berlin, Berlin, Germany; 2 Charité - Universitätsmedizin Berlin, Corporate Member of Freie Universität Berlin and Humboldt-Universität zu Berlin, Institute of Biometry and Clinical Epidemiology, Berlin, Germany; 3 Institute of Pathology, Klinikum Chemnitz gGmbH, Chemnitz, Germany; 4 Department of Oral and Maxillofacial Surgery, Fachklinik Hornheide, Münster, Germany

**Keywords:** oral squamous cell carcinoma, prognostic marker, BMI, body mass index, prognosis, surgical treatment, OSCC

## Abstract

**Objective:**

Additional prognostic factors in patients with early-stage oral squamous cell carcinoma (OSCC) may optimize disease staging by identifying patients with high-risk constellations and facilitating risk-stratified therapies that lead to improved treatment outcome. Body mass index (BMI) is an established tool that is routinely recorded in everyday clinical practice and has demonstrated prognostic relevance in various other diseases. However, sufficient evidence regarding its impact in OSCC is lacking. The aim of this study is to evaluate the prognostic significance of pretherapeutic BMI in surgically treated OSCC patients.

**Materials and methods:**

This retrospective analysis included all patients with primary OSCC who underwent surgical therapy with or without the need for adjuvant therapy at Charité – Universitätsmedizin Berlin over a seven-year period. BMI was categorized based on the World Health Organization classification and correlated with clinical outcome. Overall survival (OS) and recurrence-free survival (RFS) were examined using Kaplan-Meier curves, Cox regression analysis, and log-rank test. The hazard ratios (HR) are presented together with 95% confidence intervals (CI).

**Results:**

A total of 394 patients (male: 257 (65.2%), female: 137 (34.8%)) with a mean age of 60.0 years were included. Of these, 25 (6.3%) met the criteria for underweight, 195 (49.2%) for normal weight, 121 (30.6%) for overweight and 55 (13.9%) for obesity. Underweight patients showed a significantly lower mean OS of 46.0 months (CI: 30.9-61.2 months) and RFS of 36.5 months (CI: 24.4-48.6 months) compared to all other BMI categories. In the multivariate Cox regression for OS, a reduced risk was observed for both normal-weight (HR: 0.32 CI: 0.11-0.90, p=0.031) and overweight patients (HR: 0.17 CI: 0.05-0.59, p=0.005) relative to those who were underweight. Regarding RFS, overweight patients demonstrated a significantly reduced risk compared to underweight individuals (HR: 0.28 CI: 0.09-0.89, p=0.031).

**Conclusion:**

Our results underscore the prognostic significance of pretherapeutic BMI as an independent risk factor in OSCC, particularly highlighting the need for intensified preoperative management in underweight patients due to their compromised outcomes.

## Introduction

With an incidence rate of 389.485 and a mortality rate of 188.230 patients per year, oral squamous cell carcinoma (OSCC) represents a clinically significant malignancy in the global context (1). Despite considerable advancements in therapy, 5-year-survival rates remain poor at approximately 56%, underscoring the ongoing need for innovation in pretherapeutic risk stratification (2, 3).

Although there is a growing understanding of the influence of host-related factors - such as nutritional status and H-index (4, 5) - these are not yet incorporated into current clinical guidelines, including those issued by the German Society, and are only marginally addressed by the National Comprehensive Cancer Network (NCCN) (6, 7). Among routinely assessed parameters in clinical practice, body mass index (BMI) - calculated as the ratio of weight to height squared (8) - has garnered increasing attention. Given its established prognostic relevance in other malignancies, particularly lung and gastric cancer (9–11), interest in its potential role in OSCC has grown steadily.

To date, only a limited number of studies have examined the prognostic significance of BMI in OSCC, and their findings remain inconsistent (12–14). Both favorable and unfavorable prognostic associations have been reported. For example, poorer survival has been observed in underweight patients with locally advanced disease (12), while other studies report higher recurrence rates among overweight individuals (14). However, due to methodological limitations - such as inclusion of only selected subgroups (e.g., patients with locally advanced tumors (12)) - comparability across studies remains limited. Further research is therefore warranted to establish more robust and generalizable evidence.

Surgical resection, potentially followed by adjuvant radiotherapy with or without concurrent chemoradiotherapy, is recommended as the standard treatment for OSCC in current clinical guidelines (6, 7). In contrast, primary chemoradiotherapy plays a subordinate role. Postoperative difficulties with oral food intake commonly lead to malnutrition, which may be further exacerbated by the side effects of adjuvant chemotherapy or radiotherapy (15, 16). In this context, a preexisting state of cachexia may further exacerbate the problem, highlighting the need for pretherapeutic stratification of high-risk patients (4). Early identification could allow for timely implementation of supportive nutritional and therapeutic interventions (13).

Given the limited data on the prognostic impact of pretherapeutic BMI in surgically treated patients with OSCC, the question arises as to whether the integration of this parameter into routine clinical.

practice is warranted. This study aimed to validate the prognostic significance of pretherapeutic BMI in a representative cohort of surgically treated OSCC patients.

## Materials and methods

### Ethics statement

The Ethics Committee of the Faculty of Medicine Charité - Universitätsmedizin Berlin approved this study (EA2/028/15).

### Patient cohort

This study included all patients diagnosed with OSCC, who underwent surgical treatment including complete neck dissection at the Department of Oral and Maxillofacial Surgery, Charité – Universitätsmedizin Berlin, between 2005 and 2011. Tumor staging was conducted according to the 7^th^ edition of the American Joint Committee on Cancer (AJCC) staging manual (17). Clinical data, including tumor stage, histopathological parameters (TNM classification and tumor grade), were collected retrospectively. Additional clinical information—such as age, gender, tumor site, treatment regimen, survival time, and recurrence—was obtained from electronic medical records. Body mass index (BMI) was categorized based on the World Health Organization (WHO) classification: underweight (BMI <18.5 kg/m²), normal weight (≥18.5 to <25 kg/m²), overweight (≥25 to <30 kg/m²) and obese (≥30 kg/m²) (8).

Overall survival (OS) was defined as the time from the date of initial diagnosis to death from any cause. Patients who experienced no event were censored at the date of last contact or at the end of the observation period (31^st^ of May 2017), whichever occurred first, if the patient was still alive. In cases where the exact date of initial diagnosis was unavailable (e.g., external diagnoses), the date of surgical therapy was used as a surrogate. Recurrence-free survival (RFS) was defined as the time from completion of therapy to the occurrence of either a recurrence or death.

Survival analyses were conducted for the entire cohort as well as stratified by categorized BMI. Additionally, further analyses were performed according to the conventional subdivision into early stage (UICC I and II) and late stage tumors (UICC III and IV).

### Statistical analysis

Data were collected using Microsoft Excel (Microsoft Corporation, Redmond, USA), and statistical analyses were conducted using IBM SPSS Statistics Version 29.0.2.0 and R Version 4.0.3 (18). Categorical variables are presented as frequencies and percentages, while continuous variables are reported as means with standard deviations (SD). Group comparisons for categorical or ordinal variables were performed using the Chi-square test or Fisher’s exact test, as appropriate.

OS and RFS were analyzed using Kaplan–Meier survival estimates, with differences assessed via the log-rank test. Univariate and multivariate Cox proportional hazards regression analyses were used to identify prognostic factors for OS and RFS. All p-values are considered exploratory and are reported without adjustment for multiple comparisons; p-values less than α = 0.05 were regarded as statistically significant. Hazard ratios (HRs) and mean survival times are presented with corresponding 95% confidence intervals (CI).

## Results

### Clinicopathological features

A total of 394 patients with surgically treated OSCC were included in this study, comprising 257 males (65.2%) and 137 females (34.8%). The mean age of patients was 60.0 years, with a range from 27 to 92 years. As previously described, all patients underwent neck dissection as part of their surgical treatment (unilateral: 45.4%; bilateral: 54.6%).

In our cohort, 25 patients (6.3%) were classified as underweight, 195 (49.2%) as normal weight, 121 (30.6%) as overweight and 55 (13.9%) as obese. As shown in **Table 1**, no statistically significant correlations were found between BMI and histopathological characteristics, including grading, T stage, N stage, or UICC stage. In contrast, BMI was significantly associated with age at the time of surgery, with underweight patients presenting at a younger age (p=0.036). Univariate analysis demonstrated a significant correlation between BMI and both smoking (Cramer-V: 0.169; p=0.016) and alcohol history (Cramer-V: 0.194; p=0.003), with a higher prevalence of these factors among underweight patients.

**Table 1:** Clinicopathological characteristics of OSCC patients in relation to their pretherapeutic BMI.

	Total	BMI [kg/m^2^]				
		<18.5	18.5 - 24.9	25.0 - 29.9	≥30	
		25 (6.3%)	195 (49.2%)	121 (30.6%)	55 (13.9%)	P-value
Age		55.0 years	59.7 years	61.8 years	59.4 years	**0.036**
Sex						
Female	137 (34.8%)	10 (40.0%)	72 (37.1%)	34 (28.1%)	21 (38.9%)	0.314
Male	257 (65.2%)	15 (60.0%)	122 (62.9%)	87 (71.9%)	33 (61.1%)	
History of Smoking						
Yes	272 (75.1%)	21 (91.2%)	143 (79.9%)	74 (67.3%)	34 (68.0%)	**0.016**
No	90 (24.9%)	2 (8.7%)	36 (20.1%)	36 (32.7%)	16 (32.0%)	
History of Alcohol						
Yes	272 (75.3%)	22 (95.7%)	142 (80.2%)	71 (65.7%)	37 (69.8%)	**0.003**
No	89 (24.7%)	1 (4.3%)	35 (19.8%)	37 (34.3%)	16 (30.2%)	
Grading						
G1	31 (8.0%)	2 (8.0%)	15 (7.8%)	11 (9.3%)	3 (5.6%)	0.663
G2	279 (71.7%)	15 (60.0%)	141 (73.4%)	81 (68.6%)	42 (77.8%)	
G3	79 (20.3%)	8 (32.0%)	36 (18.8%)	26 (22.0%)	9 (16.7%)	
pT stage						
T1	174 (44.2%)	8 (32.0%)	87 (44.8%)	58 (47.9%)	21 (38.9%)	0.304
T2	144 (36.5%)	13 (52.0%)	60 (30.9%)	46 (38.0%)	25 (46.3%)	
T3	45 (11.4%)	2 (8.0%)	26 (13.4%)	13 (10.7%)	4 (7.4%)	
T4a	30 (7.6%)	2 (8.0%)	20 (10.3%)	4 (3.3%)	4 (7.4%)	
T4b	1 (0.3%)	0	1 (0.5%)	0	0	
pN stage						
N0	265 (67.3%)	19 (76.0%)	124 (63.9%)	88 (72.7%)	34 (63.0%)	0.083
N1	62 (15.7%)	2 (8.0%)	28 (14.4%)	18 (14.9%)	14 (25.9%)	
N2	67 (17.0%)	4 (16.0%)	42 (21.6%)	15 (12.4%)	6 (11.1%)	
pUICC stage						
I	132 (33.7%)	6 (24.0%)	66 (34.2%)	44 (36.7%)	16 (29.6%)	0.331
II	95 (24.2%)	9 (36.0%)	39 (20.2%)	32 (26.7%)	15 (27.8%)	
III	78 (19.9%)	4 (16.0%)	34 (17.6%)	26 (21.7%)	14 (25.9%)	
IVa	86 (21.9%)	6 (24.0%)	53 (27.5%)	18 (15.0%)	9 (16.7%)	
IVb	1 (0.3%)	0	1 (0.5%)	0	0	
Resection Status						
R0	352 (89.6%)	23 (92.0%)	168 (86.6%)	110 (91.7%)	51 (94.4%)	0.484
R1	34 (8.7%)	2 (8.0%)	19 (9.8%)	10 (8.3%)	3 (5.6%)	
R2	3 (0.8%)	0	3 (1.5%)	0	0	
Rx	4 (1.0%)	0	4 (2.1%)	0	0	
Lymphatic Invasion						
Yes	31 (16.0%)	2 (16.7%)	15 (15.6%)	11 (19.3%)	3 (10.3%)	0.761
No	163 (84.0%)	10 (83.3%)	81 (84.4%)	46 (80.7%)	26 (89.7%)	
Vascular Invasion						
Yes	5 (2.5%)	0	4 (3.8%)	1 (1.8%)	0	0.560
No	192 (97.5%)	11 (100%)	100 (96.2%)	56 (98.2%)	30 (100%)	
Extracapsular Spread						
Yes	53 (13.5%)	3 (12.5%)	32 (16.5%)	11 (9.1%)	7 (13.0%)	0.315
No	340 (86.5%)	21 (87.5%)	162 (83.5%)	110 (90.9%)	47 (87.0%)	
Adjuvant Radio(chemo)therapy						
Yes	111 (28.2%)	6 (24.0%)	63 (32.5%)	29 (24.0%)	13 (24.1%)	0.321
No	283 (71.8%)	19 (76.0%)	131 (67.5%)	92 (76.0%)	41 (75.9%)	

Bold values indicate statistical significance.

#### Survival analysis according to pretherapeutic BMI

In the entire cohort OS averaged 88.78 months (95% CI: 83.5–94.1), with a median of 106.1 months (95% CI: 85.6–127.2). RFS showed a mean of 72.9 months (95% CI: 67.3–78.4) and a median of 74.5 months (95% CI: 57.8–91.3). The 5-year overall survival rate was 65%.

**Figure 1:**
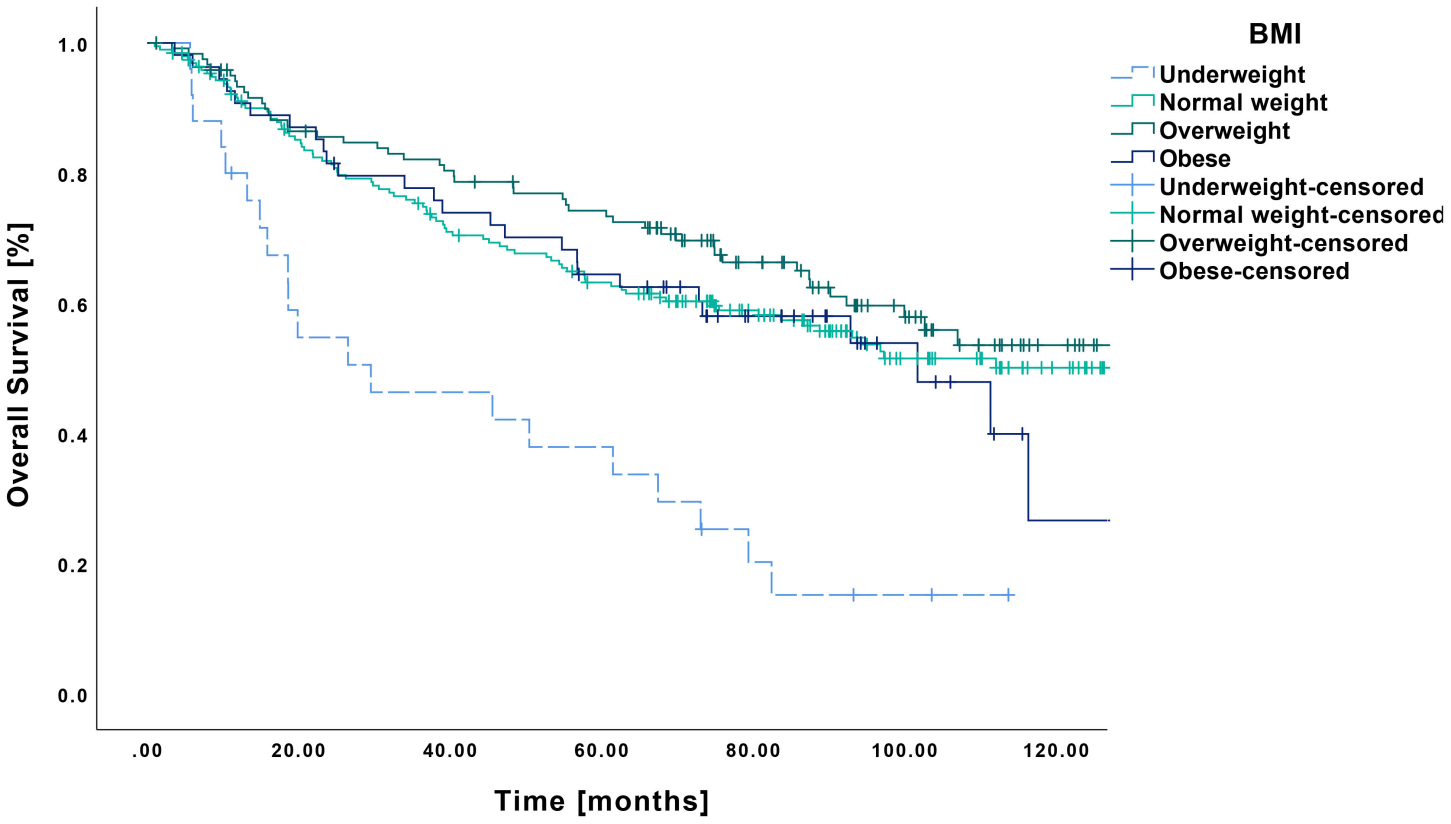
Kaplan-Meier curves showing OS in relation to pretherapeutic BMI. Patients classified as underweight (n=25) demonstrated a significantly reduced overall survival compared to those with normal weight (n=194, p<0.001), overweight (n=121, p<0.001) or obesity (n=54, p<0.001).

The Kaplan–Meier analysis revealed a mean OS of 46.0 months (CI: 30.9-61.2 months) in underweight patients, which was significantly lower than that of normal weight (89.4 months CI: 81.8-97.0 months, p<0.001), overweight (97.4 months CI: 88.4-106.5 months, p<0.001) and obese patients (83.0 months CI: 70.3-95.8 months, p<0.001) (**Figure 1**). The 5-year survival rates, in the order mentioned before, were 39%, 64%, 74%, and 64%, respectively.

The multivariate Cox regression model, which included clinical and histopathological characteristics, identified BMI and resection status as independent prognostic factor for OS (**Table 2**). Specifically, patients with normal weight demonstrated a reduced risk with a HR of 0.32 (CI: 0.11-0.90, p=0.031) and overweight patients with an HR of 0.17 (CI: 0.05-0.59, p=0.005), all compared to underweight patients. In contrast, no significant risk reduction was observed in obese patients (HR 0.35 CI: 0.10-1.21, p=0.099).

**Table 2:** Univariate and multivariate Cox regression analysis for OS.

	Univariate analysis			Multivariate analysis		
	HR	95%-CI	P-value	HR	95%-CI	P-value
BMI						
Underweight	1			1		
Normal weight	0.35	0.22 – 0.58	**<0.001**	0.32	0.11 – 0.90	**0.031**
Overweight	0.29	0.17 – 0.49	**<0.001**	0.17	0.05 – 0.59	**0.005**
Obese	0.41	0.23 – 0.72	**0.002**	0.35	0.10 – 1.21	0.099
Sex						
male	1			1		
female	0.93	0.68 – 1.27	0.643	0.89	0.43 – 1.80	0.736
History of Smoking						
Yes	1			1		
No	0.52	0.33 – 0.79	**0.002**	0.86	0.39 – 1.87	0.699
History of Alcohol						
Yes	1			1		
No	0.76	0.52 – 1.11	0.157	1.14	0.56 – 2.33	0.720
Grading						
G1	1			1		
G2	1.81	0.95 – 3.45	0.073	3.48	0.46 – 26.29	0.227
G3	2.89	1.46 – 5.75	**0.002**	2.19	0.25 – 19.50	0.483
pT Stage						
T1	1			1		
T2	2.41	1.70 – 3.44	**<0.001**	0.35	0.10 – 1.17	0.088
T3	3.94	2.53 – 6.14	**<0.001**	0.92	0.24 – 3.55	0.901
T4a	3.29	1.92 – 5.64	**<0.001**	1.52	0.13 – 17.51	0.737
T4b	0	–	0.961	–	–	–
pN Stage						
N0	1			1		
N1	1.73	1.16 – 2.56	**0.007**	0.58	0.13 – 1.17	0.473
N2	3.03	2.13 – 4.31	**<0.001**	1.67	0.14 – 19.72	0.682
UICC Stage						
I	1			1		
II	2.41	1.52 – 3.80	**<0.001**	2.95	0.74 – 11.74	0.125
III	3.37	2.14 – 5.31	**<0.001**	3.80	0.72 – 20.24	0.117
IVa	4.78	3.09 – 7.39	**<0.001**	1.87	0.13 – 27.19	0.646
IVb	0	–	0.962			
Resection Status						
R0	1			1		
R1	3.15	2.09 – 4.77	**<0.001**	6.10	1.60 – 23.34	**0.008**
R2	5.64	1.38 – 22.95	**0.016**	–	–	**<0.001**
Rx	1.0	0.14 – 7.16	1.0	4.48	0.38 – 53.49	0.236
Lymphatic Invasion						
Yes	1.96	1.14 – 3.37	**0.015**	2.64	1.0 – 6.98	0.051
No	1			1		
Vascular Invasion						
Yes	1.29	0.41 – 4.09	0.668	0.83	0.10 – 7.11	0.870
No	1			1		
Extracapsular Spread						
Yes	2.99	2.09 – 4.27	**<0.001**	3.28	0.93 – 11.52	0.064
No	1			1		
Adjuvant Radio(chemo)therapy						
Yes	2.21	1.63 – 2.99	**<0.001**	0.55	0.19 – 1.54	0.254
No	1			1		

Bold values indicate statistical significance.

**Figure 2:**
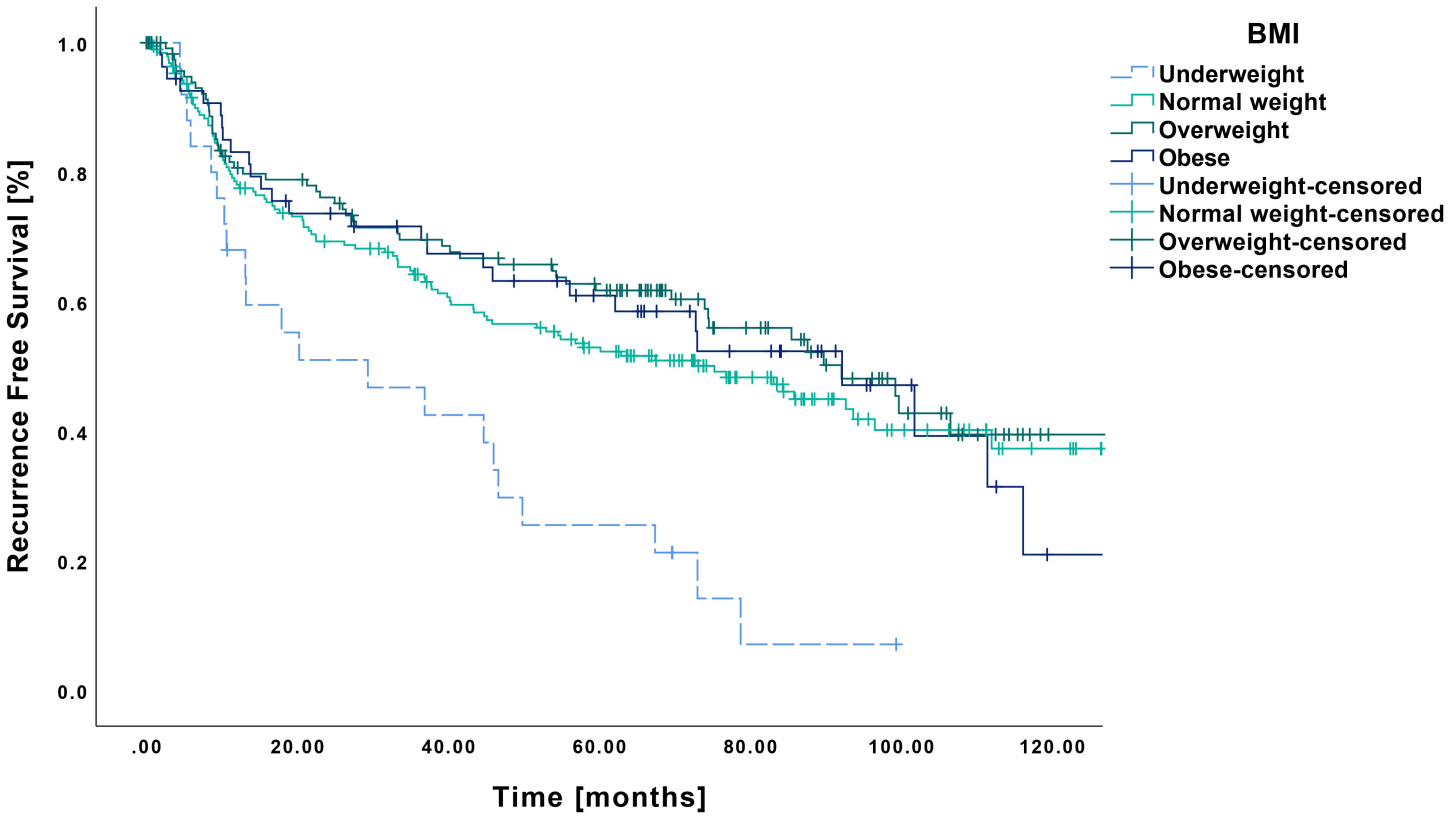
Kaplan-Meier curves showing RFS in relation to pretherapeutic BMI. RFS was significantly reduced in underweight patients (n=25) compared to those with normal weight (n=194, p<0.001), overweight (n=121, p<0.001) or obesity (n=54, p<0.001).

Regarding RFS, a similar pattern emerged, with underweight patients showing significantly poorer outcomes (**Figure 2**). Their mean RFS was 36.5 months (CI: 24.4-48.6 months), which differed significantly from that of normal weight (73.0 months CI: 65.0-81.0 months, p<0.001), overweight (78.3 months CI: 68.7-87.8 months, p<0.001) and obese patients (74.4 months, CI: 60.5-88.3 months, p<0.001).

In the multivariate analysis, underweight and R2 resection emerged as independent risk factors for RFS. Overweight patients demonstrated a prognostic advantage over underweight patients, with a HR of 0.28 (CI: 0.09-0.89, p=0.031), whereas no significant differences were observed for normal weight (HR: 0.51 CI: 0.19-1.38, p=0.188) and obese patients (HR 0.44 CI: 0.13-1.43, p=0.170). A detailed overview is provided in **Table 3**.

**Table 3:** Univariate and multivariate Cox regression analysis for RFS.

	Univariate analysis			Multivariate analysis		
	HR	95%-CI	P-value	HR	95%-CI	P-value
BMI						
Underweight	1			1		
Normal weight	0.44	0.28 – 0.71	**<0.001**	0.51	0.19 – 1.38	0.188
Overweight	0.37	0.22 – 0.61	**<0.001**	0.28	0.09 – 0.89	**0.031**
Obese	0.43	0.24 – 0.75	**0.003**	0.44	0.13 – 1.43	0.170
Sex						
male	1			1		
female	1.02	0.76 – 1.36	0.889	0.884	0.42 – 2.16	0.793
History of Smoking						
Yes	1			1		
No	0.55	0.37 – 0.80	**0.002**	0.88	0.42 – 1.86	0.745
History of Alcohol						
Yes	1			1		
No	0.81	0.57 – 1.16	0.258	1.32	0.66 – 2.66	0.434
Grading						
G1	1			1		
G2	1.97	1.04 – 3.75	**0.038**	3.23	0.43 – 24.25	0.253
G3	3.25	1.64 – 6.42	**<0.001**	3.17	0.37 – 26.92	0.290
pT Stage						
T1	1			1		
T2	2.19	1.57 – 3.05	**<0.001**	0.88	0.28 – 2.81	0.831
T3	3.41	2.23 – 5.21	**<0.001**	1.76	0.47 – 6.52	0.401
T4a	3.37	2.04 – 5.55	**<0.001**	2.82	0.20 – 40.53	0.446
T4b	5.36	0.74 – 38.86	0.097	–	–	–
pN Stage						
N0	1			1		
N1	1.58	1.09 – 2.31	**0.016**	0.84	0.21 – 1.3.41	0.812
N2	2.53	1.81 – 3.54	**<0.001**	1.27	0.10 – 15.81	0.852
UICC Stage						
I	1			1		
II	2.10	1.38 – 3.21	**<0.001**	1.60	0.42 – 6.02	0.491
III	2.87	1.89 – 4.38	**<0.001**	2.17	0.43 – 10.90	0.348
IVa	3.86	2.58 – 5.78	**<0.001**	1.70	0.10 – 29.53	0.715
IVb	6.45	0.88 – 47.21	0.066			
Resection Status						
R0	1			1		
R1	3.22	2.18 – 4.77	**<0.001**	3.29	0.95 – 11.45	0.061
R2	5.70	1.81 – 18.00	**0.003**	13.04	1.22 – 139.21	**0.033**
Rx	0.72	0.10 – 5.12	0.793	2.60	0.23 – 29.52	0.444
Lymphatic Invasion						
Yes	1.86	1.12 – 3.11	**0.017**	1.93	0.78 – 4.77	0.156
No	1			1		
Vascular Invasion						
Yes	1.66	0.61 – 5.52	0.326	3.51	0.64 – 19.22	0.147
No				1		
Extracapsular Spread						
Yes	2.44	1.73-3.45	**<0.001**	2.28	0.66 – 7.93	0.195
No	1			1		
Adjuvant Radio(chemo)therapy						
Yes	2.05	1.54 – 2.74	**<0.001**	0.60	0.23 – 1.59	0.307
No	1			1		

Bold values indicate statistical significance.

#### Survival analysis according to pretherapeutic BMI and stage of disease

Additional analyses were conducted stratified by early- (UICC I and II) and late-stage disease (UICC III and IV) to investigate potential stage-specific differences in the prognostic relevance of BMI.

### Early-stage OSCC (UICC I and II)

In early-stage patients (n=227), the mean OS was 102.9 months (CI: 96.6-109.1 months), with a 5-year survival rate of 77%, respectively. In this cohort, RFS averaged 87.1 months (CI: 80.0-94.2 months).

**Figure 3:**
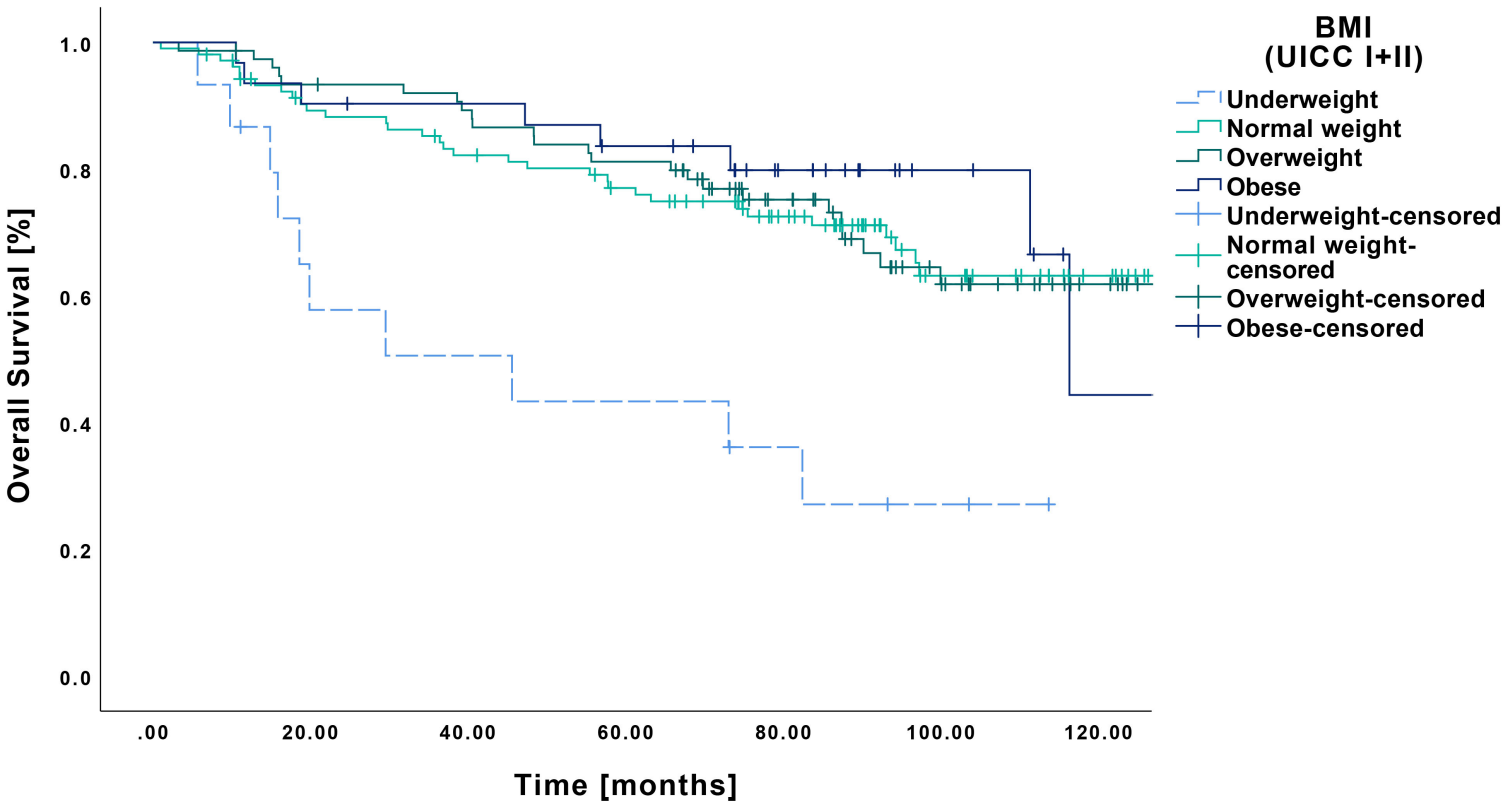
Kaplan-Meier curves showing OS of early-stage OSCC (UICC I and II) in relation to pretherapeutic BMI. Underweight patients (n=15) showed a significantly lower OS compared to normal weight (n=105, p<0.001), overweight (n=76, p<0.001), and obese patients (n=31, p<0.001).

In terms of OS in relation to pretherapeutic BMI, underweight patients demonstrated lower survival compared to all other BMI categories (**Figure 3**). The underweight cohort showed a mean OS of 54.9 months (CI: 32.7-77.1 months), resulting in statistically significant differences when contrasted with normal weight (104.3 months CI: 95.0-113.7 months, p<0.001), overweight (103.7 months CI: 94.4-113.7 months, p<0.001) and obese patients (105.1 months CI: 90.5-119.7 months, p<0.001).

**Figure 4:**
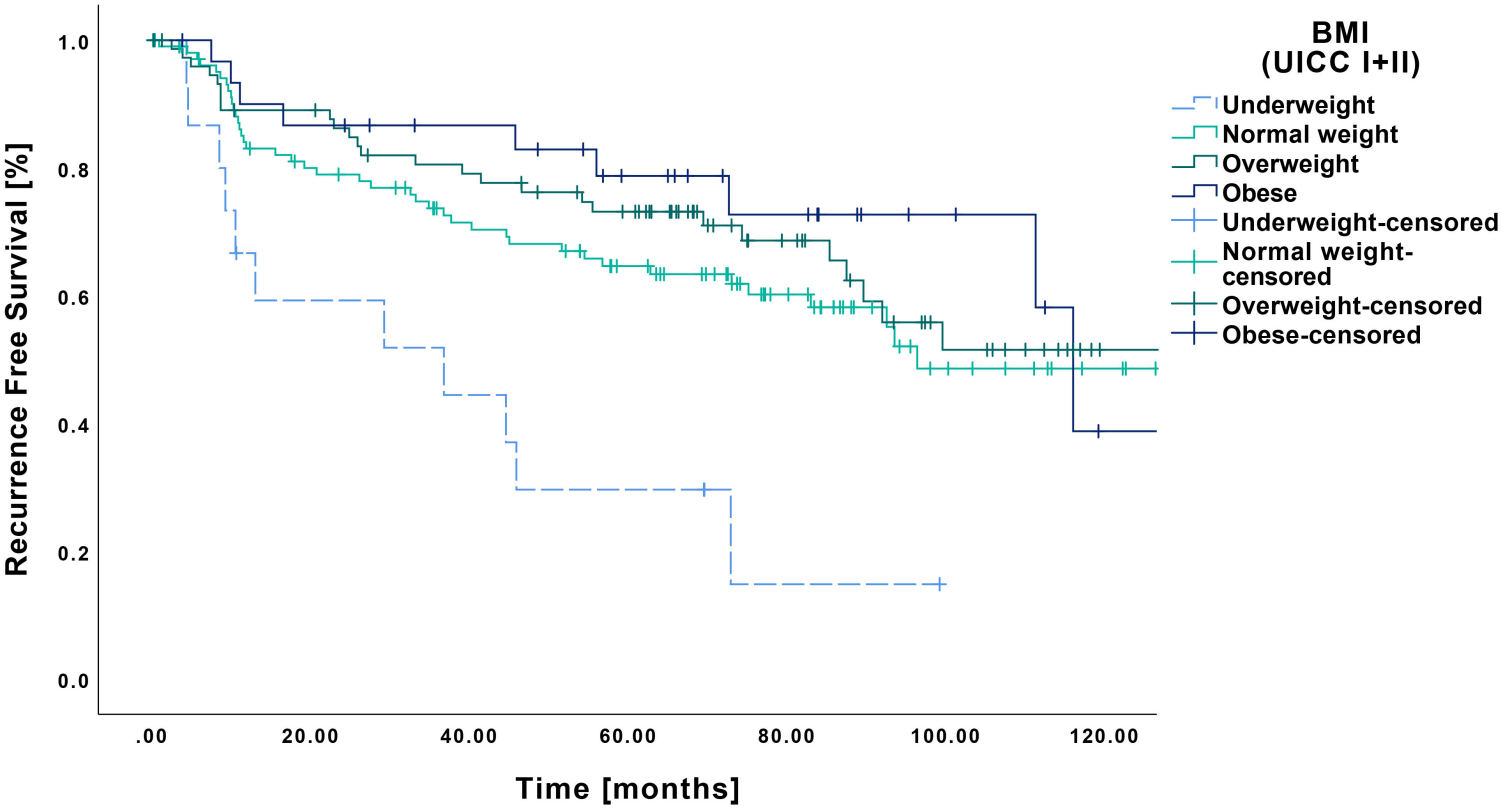
Kaplan-Meier curves showing RFS of early-stage OSCC (UICC I and II) in relation to pretherapeutic BMI. Underweight patients (n=15) demonstrated a significantly shorter RFS compared to normal weight (n=105, p=0.002), overweight (n=76, p=0<0.001) and obese patients (n=31, p<0.001).

A similar pattern was observed for RFS, with the underweight group showing inferior outcomes compared to all other BMI categories (**Figure 4**). Specifically, the underweight patients had a mean RFS of 40.6 months (CI: 22.9-58.3 months), which differed significantly from that of the normal weight (86.1 months CI: 75.4-96.8 months, p=0.002), overweight (91.0 months CI: 79.9-102.0 months, p<0.001) and obese groups (98.3 months CI: 81.5-115.0 months, p<0.001).

### Late-stage OSCC (UICC III and IV)

In the cohort of late-stage tumors (n=167), the mean OS was 69.1 months (CI: 61.0-77.2 months), with a RFS of approximately 53.6 months (CI: 45.7-61.4 months). The 5-year survival rate was 49%.

**Figure 5:**
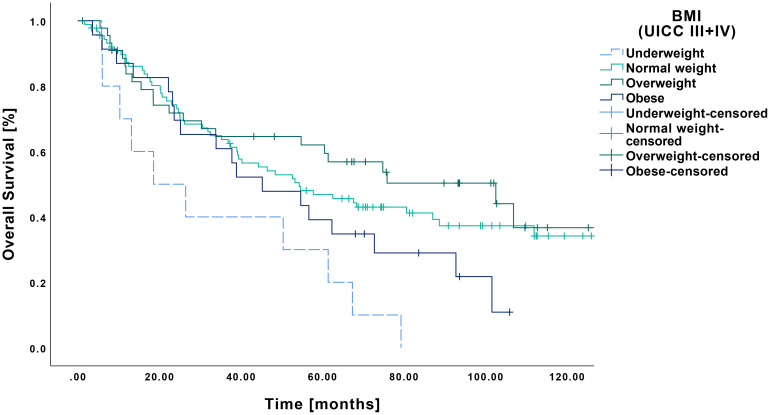
Kaplan-Meier curves showing OS of late-stage OSCC (UICC III and IV) in relation to pretherapeutic BMI. RFS was significantly reduced in underweight patients (n=10) compared to those with normal weight (n=89, p=0.006) and overweight (n=45, p=0.002), whereas no significant difference was observed when compared to obese patients (n=23, p=0.080).

Regarding OS according to BMI, underweight patients showed a mean survival of 33.9 months (CI: 16.5-51.4 months), which differed significantly from normal weight (70.8 months CI: 59.7-71.8 months, p=0.006) and overweight patients (79.7 months CI: 63.1-96.3 months, p=0.002) (**Figure 5**). In contrast, no significant difference was observed compared to obese patients (53.4 months CI: 38.8-67.9 months, p=0.080), although a clear trend was apparent.

**Figure 6:**
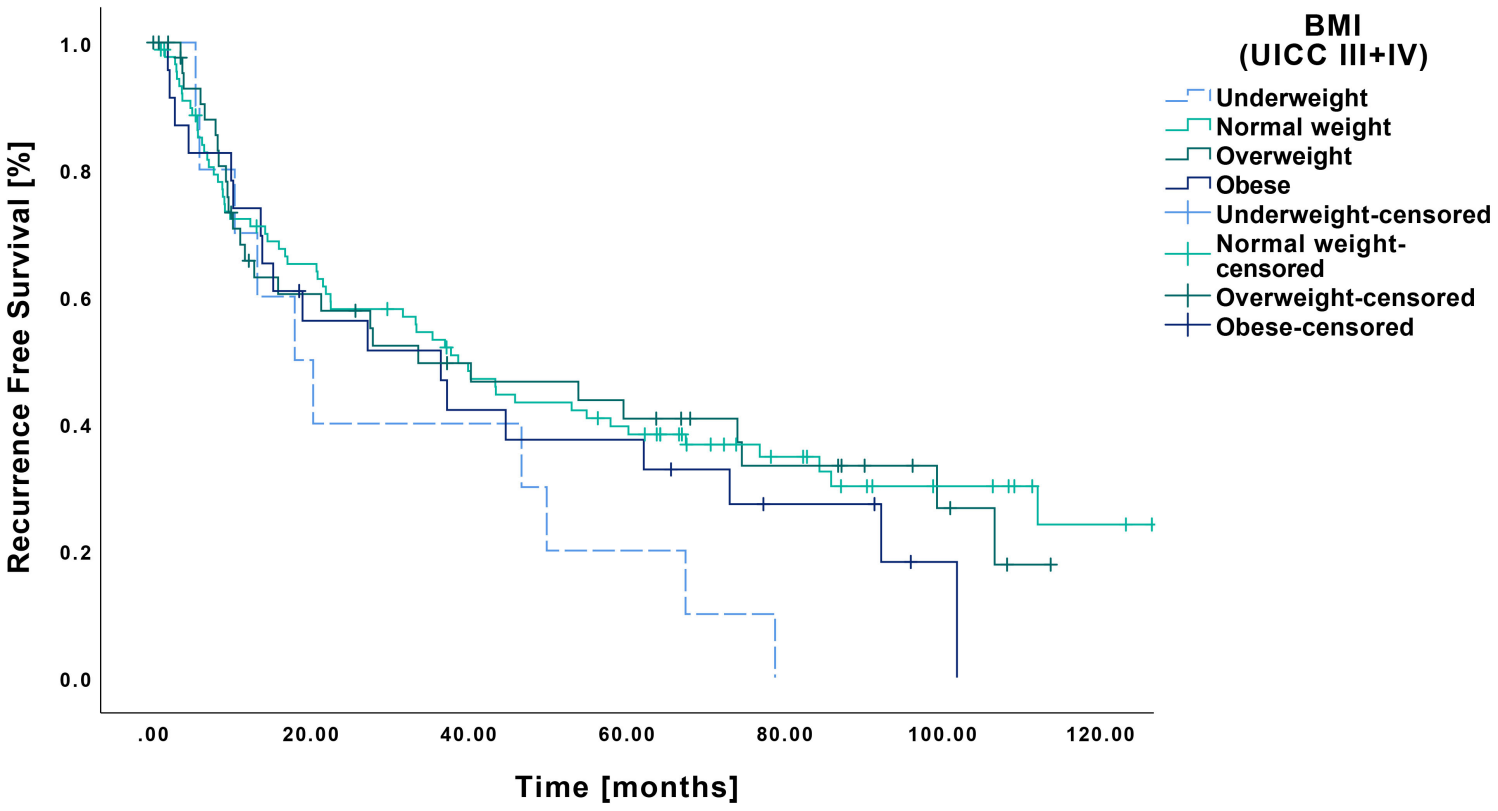
Kaplan-Meier curves showing RFS of late-stage OSCC (UICC III and IV) in relation to pretherapeutic BMI. Patients classified as underweight (n=10) demonstrated no significant differences regarding RFS compared to those with normal weight (n=89, p=0.073), overweight (n=45, p=0.093) or obesity (n=23, p=0.247).

RFS among underweight patients with late-stage tumors was 31.5 months (CI: 14.9-48.2 months). No significant differences were observed in comparison to normal weight (57.8 months CI: 46.5-69.0 months, p=0.073), overweight (52.6 months CI: 38.6-66.8 months, p=0.093) or obese patients (44.7 months CI: 28.4-61.0 months, p=0.247) (**Figure 6**).

## Discussion

The aim of this study was to explore the prognostic significance of pretherapeutic BMI in a representative cohort OSCC patients OSSC that were treated surgically with or without the need for adjuvant therapy.

In the present cohort, no significant associations were found between BMI categories and histopathological characteristics such as tumor stage, grading, lymphatic or vascular invasion. These findings are consistent with previously published cohorts, which also reported no influence of pretherapeutic BMI on histological phenotype (13, 19). In contrast, significant correlations were observed between BMI and age, smoking, and alcohol consumption in our cohort. Underweight patients tended to present at a younger age and predominantly exhibited classic risk factors such as tobacco and alcohol use. This excessive risk profile may contribute to earlier disease onset, and comorbidities associated with active smoking and alcohol abuse may explain the lower BMI observed. However, considering other studies that reported no association between BMI, age, and risk behavior (13, 19), these interpretations remain speculative.

In our overall cohort, underweight patients showed a significantly worse OS and RFS compared to all other BMI categories. Multivariate analysis revealed a reduced risk for normal-weight and overweight patients in comparison to those who were underweight.

Some studies have reported similar findings (12, 13). For example, Chang et al. observed a poorer prognosis among underweight patients in a comparable cohort of 320 surgically treated individuals (13). Based on their results, the authors proposed preoperative nutritional optimization as a potential intervention (13). However, a key limitation of their study was the BMI classification: all patients with a BMI above 25 were grouped together, with no distinction made between overweight and obese categories (13). This lack of granularity limits the comparability with other studies. Moreover, when evaluating the generalizability of the present study’s findings, the markedly high proportion of male patients (91.9%) must also be taken into consideration (13). In contrast, Wang et al. reported a higher recurrence rate and poorer prognosis among obese patients, although underweight individuals were found to experience increased rates of postoperative complications (14). Other authors, however, have proposed that BMI has no significant impact on prognosis, instead emphasizing the relevance of other host-related factors (5).

In patients with locally advanced disease, OS was significantly higher in both normal-weight and overweight individuals, while RFS showed a clear trend in favor of these groups. In a single-institution study, Ma et al. reported a prognostic advantage for overweight patients compared to those of normal weight (20). However, in contrast to the present study, their cohort included only patients treated with primary radio chemotherapy (20). The high rate of metabolic response observed among overweight and obese patients may have contributed to these findings (20). Furthermore, their study included tumors from the entire head and neck region. Given the superior response rates of oropharyngeal squamous cell carcinomas to primary radio chemotherapy (21), the comparability with the present, clearly defined OSCC cohort is limited. Another possible explanation could be the so-called obesity paradox, although its very existence remains a subject of ongoing debate (22). This theory suggests that, despite the well-established negative health effects of obesity, a survival advantage has been observed in certain chronic diseases and cancers (22). However, some authors attribute these findings, at least in part, to methodological limitations in the underlying studies (22). A potential improvement could be the inclusion of additional factors, such as body composition (23).

Zhao et al. also demonstrated an inferior prognosis in underweight patients compared to overweight and obese individuals (12). However, it is important to note the inconsistent definition of BMI categories in their study, with the threshold between overweight and obesity set at 27.5 (12). A standardized classification and subsequent pooling of data would be desirable to enable more robust evaluations in future analyses.

In contrast to the aforementioned studies, obese patients in our cohort did not demonstrate a survival advantage over underweight individuals. While this does not indicate an axiomatically inferior prognosis compared to normal-weight and overweight patients, a general disadvantage remains evident. A similar finding was reported by Iyengar et al. in their cohort of early-stage tongue cancer patients, where obese individuals had poorer outcomes compared to those with normal weight (19). One important distinction compared to other studies is the relatively low rate of adjuvantly treated patients (n=8) (19). As previously discussed, this subgroup may benefit more distinctly from treatment, potentially contributing to the improved outcomes observed in obese patients elsewhere (20).

In light of the existing latency between diagnosis and the actual surgery — as is the case, for example, with CAD/CAM planning — some authors are exploring the potential for dedicated preoperative patient preparation (24). With the increasing establishment of neoadjuvant therapeutic approaches (25, 26), this potential continues to grow. For both OSCC and other malignancies, such as esophageal carcinoma, promising data have emerged regarding this patient preparation approach referred to as prehabilitation (24, 27). For example, optimal management of diabetes mellitus and the use of immunonutrition have been associated with improved clinical outcomes, such as a reduction in wound infections (28, 29). Our data identify a new subgroup that requires dedicated preoperative optimization — regardless of whether the reduced BMI represents a surrogate marker for an underlying disease or indicates malnutrition per se. In this context, optimization of underlying comorbidities, targeted speech therapy, physiotherapy, and improvement of nutritional intake according to the underlying cause may be beneficial.

In our cohort, multivariate analysis revealed - besides the already discussed effects of BMI - a significant impact of R1- or R2 resection on OS and RFS. Several studies have previously demonstrated the negative prognostic impact of incomplete resection in OSCC (30). Current clinical guidelines recommend adjuvant therapy in the presence of such intraoperative risk factors, not least for this reason (6, 7). Some authors even suggest a direct association between resection status and the histopathological aggressiveness of the tumor, indicating that an incomplete resection may reflect an inherently more invasive disease biology (31).

Our study is based on a large, well-defined cohort of patients with OSCC who underwent curatively intended surgery followed by adjuvant therapy where indicated. Limitations of this study include its retrospective design, the use of the 7th edition of the AJCC classification system, and incomplete documentation of certain histopathological features such as perineural invasion or depth of invasion. Moreover, precise data on the cause of death were not available, thereby limiting the ability to draw causal conclusions. Additionally, the reasons for foregoing adjuvant therapy were not always provided. Given that BMI could potentially influence treatment decisions and, consequently, patient prognosis, prospective studies with precise correlation between BMI, therapeutic choices and outcomes are needed.

In summary, our results underscore the prognostic significance of pretherapeutic BMI as an independent risk factor in OSCC. Additional studies investigating the direct effect of prehabilitation on clinical outcomes in this patient subgroup are warranted.

## Data Availability

The raw data supporting the conclusions of this article will be made available by the authors, without undue reservation.
